# The impact of muscle mass loss and deteriorating physical function on prognosis in patients receiving hemodialysis

**DOI:** 10.1038/s41598-021-01581-z

**Published:** 2021-11-16

**Authors:** Mineaki Kitamura, Takahiro Takazono, Kosei Yamaguchi, Satoko Notomi, Kenji Sawase, Takashi Harada, Satoshi Funakoshi, Hiroshi Mukae, Tomoya Nishino

**Affiliations:** 1 Nagasaki Renal Center, Nagasaki, Japan; 2 Department of Nephrology, Nagasaki University Graduate School of Biomedical Sciences, Nagasaki, Japan; 3 Department of Infectious Diseases, Nagasaki University Graduate School of Biomedical Sciences, Nagasaki, Japan; 4 Department of Respiratory Medicine, Nagasaki University Hospital, Nagasaki, Japan; 5 Department of Respiratory Medicine, Nagasaki University Graduate School of Biomedical Sciences, Nagasaki, Japan

**Keywords:** Renal replacement therapy, Malnutrition, Geriatrics

## Abstract

Muscle mass loss and worsening physical function are crucial issues in patients receiving hemodialysis (HD). However, few studies have investigated the association between temporal changes in muscle mass and physical function in a large number of HD patients. We examined 286 patients receiving HD (males, 58%; age, 66.8 ± 13.0 years) at a single center, and calculated the percent changes in psoas muscle mass index (%PMI) using computed tomography over two screenings, once per year (July 2011–June 2013). Physical function was evaluated using the Eastern Cooperative Oncology Group Performance Status (ECOG-PS) (range 0–4). The observation period was from July 2012 to June 2021. The median %PMI was -9.5%, and those with the lowest quartile of %PMI (< −20.5%) showed a significantly poor prognosis compared with other patients (p < 0.001). Multivariable logistic regression analysis revealed that these patients tended to have decreased physical function (ECOG-PS 2–4) [odds ratio (OR): 2.46, p < 0.001] and albumin levels (OR: 0.22, p = 0.007). Multiple-factor-adjusted Cox regression analyses showed that %PMI (hazard ratio: 0.99, p = 0.004) and each ECOG-PS stage (1–4 vs. 0) (p < 0.01) were associated with mortality. Augmenting physical activities in daily life and serum albumin levels should be considered to maintain muscle mass and improve the prognosis of patients receiving HD.

## Introduction

Skeletal muscle loss in patients receiving hemodialysis (HD) lowers their quality of life and worsens activities of daily living and physical function. Moreover, it has been associated with all-cause mortality^1–4^. Therefore, effective and practical countermeasures are urgently needed. However, uncertainties regarding the factors related to muscle mass loss remain. Patients with end-stage renal disease are more likely to have decreased muscle mass due to the effects of protein energy wasting. Moreover, patients requiring dialysis tend to have an advanced age^5^, which means that they also experience aging-related muscle loss.

Muscle mass is generally assessed using body composition, dual-energy X-ray absorptiometry, mid-arm muscle circumference, and the sum of skinfold thickness^6^; however, it remains difficult to assess a large number of patients over time in routine medical care. Recently, several studies have used an alternative method to assess muscle mass in patients receiving HD, using the psoas muscle index (PMI), which is calculated based on the transverse area of the psoas muscle at the level of the third lumbar spine, identified via abdominal computed tomography (CT)^7–9^. The PMI is highly reproducible and considered a useful tool because it reflects the skeletal muscle mass of the whole body^7,10^.

Our aims included the following: (i) to investigate the relationship between annual changes in the PMI and physical function in daily life; (ii) to clarify the factors associated with muscle mass loss to improve clinical practice for patients receiving HD; and (iii) to evaluate the effects of muscle mass loss and physical function on all-cause mortality.

## Results

### Patient background

We included 286 patients (age, 66.8 ± 13.0 years; males, 58%) who received HD continuously at the Nagasaki Renal Center. All participants underwent a couple of routine birth month examinations, including blood examinations and abdominal CT, from July 1, 2011, to June 30, 2013. The patient flow is shown in Supplementary Fig. 1. The participants had a history of ischemic heart disease (34%), stroke (26%), arteriosclerosis (17%), and diabetes mellitus (34%). Other patient characteristics are shown in Table 1 and Supplementary Table 1.

**Table 1:** Patient background.

	At the time of entry (N = 286)	One year after (N = 286)	P value
Age (years)	65.8 ± 13.0	66.8 ± 13.0	
Male (%)	58.0	58.0	
Dialysis vintage (months)	61 (24–126)	73 (36–138)	
Dialysis time (h)	4 (3–4)/3.73 ± 0.58	4 (3–4)/3.70 ± 0.58	0.03
CTR (%)	51.6 ± 5.6	52.0 ± 5.3	0.051
Dry weight (kg)	53.1 ± 10.8	52.7 ± 10.9	0.02
Body mass index (kg/m^2^)	21.1 ± 3.3	20.9 ± 3.4	0.02
Systolic blood pressure (mmHg)	150 ± 24	150 ± 27	0.94
Hb (g/dL)	10.9 ± 1.4	10.5 ± 1.2	< 0.001
Ferritin^a^ (ng/mL)	64 (23–185)	60 (14–130)	< 0.001
TSAT (%)	23 (16–32)	20 (13–29)	0.008
Alb (g/dL)	3.6 ± 0.4	3.5 ± 0.5	< 0.001
cCa (mg/dL)	9.2 ± 0.7	9.2 ± 0.6	0.80
P (mg/dL)	5.6 ± 1.6	5.4 ± 1.7	0.02
Intact-PTH^a^ (pg/mL)	79 (29–157)	74 (25–151)	0.66
ALP^a^ (IU/L)	247 (191–336)	252 (195–320)	0.58
BUN (mg/dL)	69 ± 17	64 ± 18	< 0.001
Cr (mg/dL)	10.6 ± 3.3	10.3 ± 3.2	0.02
Total cholesterol (mg/dL)	161 ± 37	162 ± 39	0.66
Triglycerides (mg/dL)	92 (67–134)	87 (65–117)	0.09
CRP^a^ (mg/dL)	0.16 (0.07–0.44)	0.16 (0.06–0.22)	0.02
KT/V	1.34 ± 0.41	1.49 ± 0.42	< 0.001
ECOG-PS 0 (%)	64	53	< 0.001
ECOG-PS 1 (%)	7	10	
ECOG-PS 2 (%)	14	15	
ECOG-PS 3 (%)	9	13	
ECOG-PS 4 (%)	6	9	

Paired-t-test, Wilcoxon sum test, and McNemar test were used for analysis.

*CTR* cardiothoracic ratio, *Hb* hemoglobin, *TSAT* transferrin saturation, *Alb* albumin, *cCa* corrected calcium, *P* phosphate, *PTH* parathyroid hormone, *ALP* alkaline phosphatase, *BUN* blood urea nitrogen, *Cr* creatinine, *CRP* C-reactive protein, *PS*, *ECOG-PS* Eastern Cooperative Oncology Group performance status.

^a^Median (interquartile range).

### PMI and physical function of the participants

**Figure 1:**
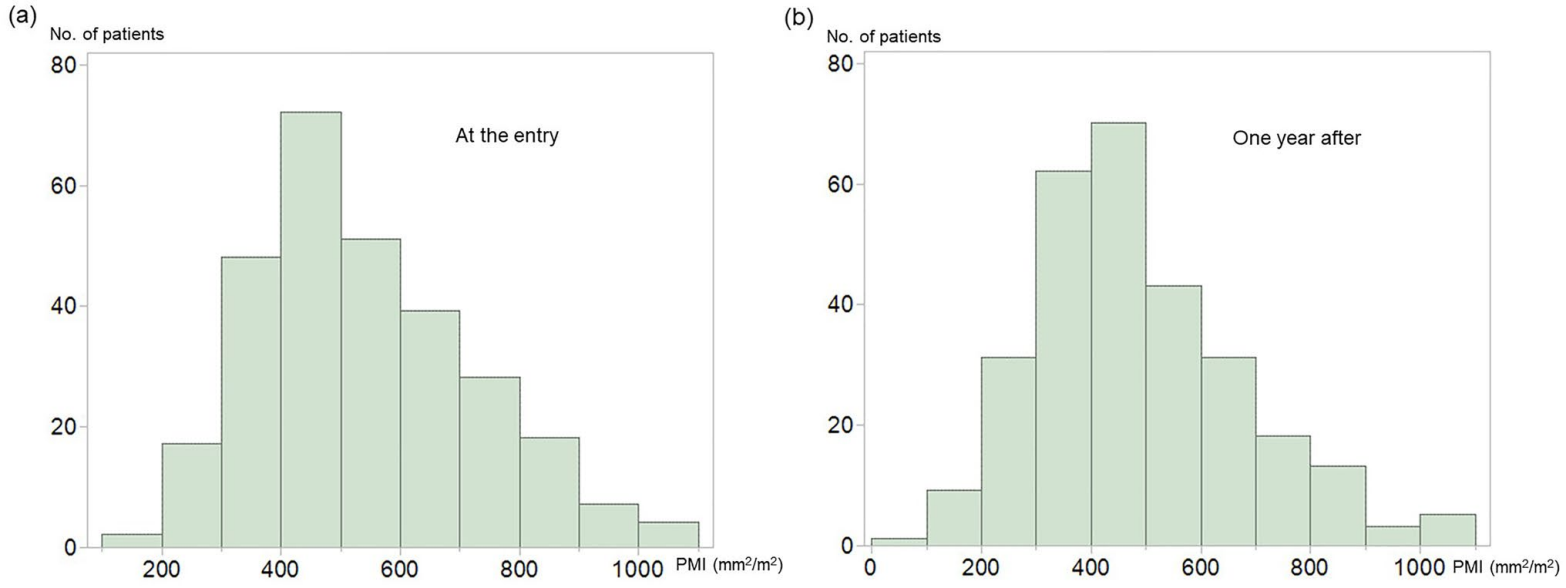
**(a)** Histogram of the psoas muscle index at entry. **(b)** Histogram of the psoas muscle index 1 year after entry. As shown in the two histograms, the patients in this study experienced 9.5% of muscle mass loss in 1 year. Statistical analyses were performed using the JMP Pro 15.0.0 (3903308).

The median PMI in the first year was 509 mm^2^/m^2^ (males, 589 mm^2^/m^2^; females, 430 mm^2^/m^2^), and that in the second year was 464 mm^2^/m^2^ (males, 532 mm^2^/m^2^; females, 371 mm^2^/m^2^). The histograms of the PMI are shown in Fig. 1A,B.

**Figure 2:**
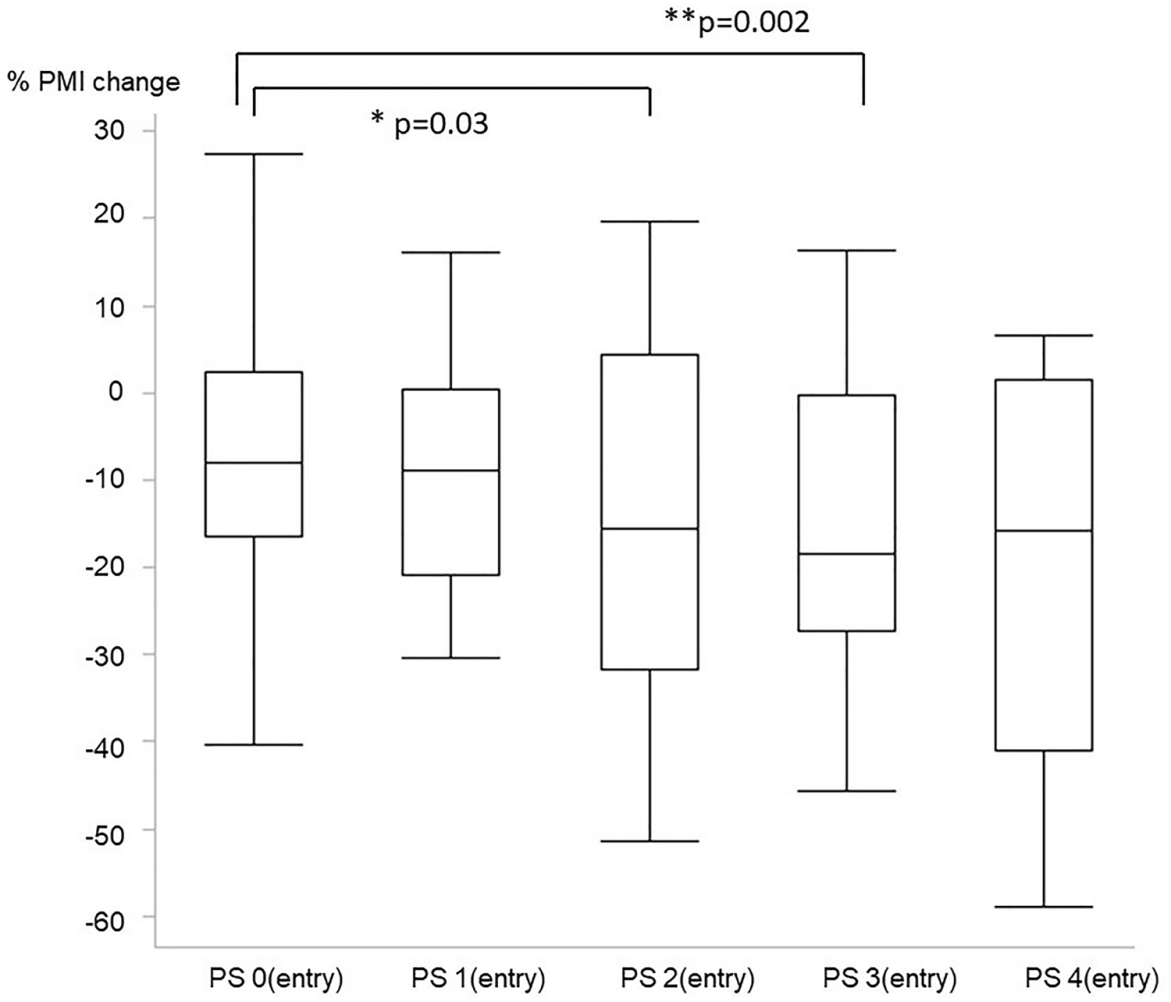
Association between percent change in the psoas muscle (%PMI) and the Eastern Cooperative Oncology Group Performance Status (ECOG-PS) scale at entry. PS: Eastern Cooperative Oncology Group Performance Status. Patients with ECOG-PS 2 and 3 at entry had greatly decreased muscle mass compared to those with ECOG-PS 0. Statistical analyses were performed using the JMP Pro 15.0.0 (3903308).

Percent changes in the PMI (%PMI), according to their physical function categorized by the Eastern Cooperative Oncology Group Performance Status (ECOG-PS) (range 0–4), are shown in Table 2. Compared with %PMI of patients with ECOG-PS 0 (fully active), that of the patients categorized as ECOG-PS 2 and 3 was extremely low (Fig. 2). Moreover, the ECOG-PS 1 year after inclusion showed the same tendency with a linear decline in each class (PS 0, −6.7%; PS 1, −11.8%; PS 2, −15.4%; PS 3, −21.7%; PS 4, −20.1%); data are shown in Supplementary Fig. 2. Furthermore, %PMI was divided into quartiles [Q1 (> + 1.8%), Q2 (−9.5% to 1.8%), Q3 (−20.5% to −9.5%), and Q4 (< −20.5%)], and the correlation among these four groups and the annual changes in blood examinations, dry weight, and cardiothoracic rate were evaluated. The annual changes in dry weight and serum albumin levels tended to be lower in accordance with the quartile of %PMI (Table 3). In terms of the association between %PMI and patient’s background at inclusion, no clear tendencies were observed among Q1–Q4 (Supplementary Table 2).

**Table 2:** Transition of the psoas muscle index by the performance status at the entry.

	At the time of the entry	One year after the entry	% decrease of PMI	P value entry point vs one year after
Total^a^ (n = 286)	509 (409–657)	464 (342–596)	−9.5 (−20.1 to 1.8)	< 0.001
ECOG-PS0^a^ (n = 183)	571 (436–715)	502 (401–643)	−8.1 (−16.5 to 2.3)	< 0.001
ECOG-PS1^a^ (n = 21)	507 (412–607)	479 (358–494)	−8.8 (−20.8 to 0.4)	0.002
ECOG-PS2^a^ (n = 39)	447 (360–564)	360 (288–497)	−15.5 (−31.7 to 4.4)	< 0.001
ECOG-PS3^a^ (n = 27)	424 (347–525)	342 (268–437)	−18.5(−27.2 to −0.3)	< 0.001
ECOG-PS4^a^ (n = 16)	416 (345–653)	337 (199–568)	−15.8 (−41.0 to 1.4)	0.02

Wilcoxon sum test was used for analysis.

*PMI* psoas muscle mass index, *ECOG-PS* Eastern Cooperative Oncology Group performance status.

^a^Median (interquartile range).

**Table 3:** Association between the quartile of psoas mass index and parameter change in one year.

	Q1 (n = 71)	Q2 (n = 72)	Q3 (n = 72)	Q4 (n = 71)	P value
ΔCTR^a^ (%)	0 (−1 to 2)	1 (−1 to 3)	1 (−1 to 2)	0 (−1 to 2)	0.45
ΔDW^a^ (kg)	0.3 (−0.7 to 1.7)	0 (−0.7 to 1.4)	0 (−1.6 to 1.5)	–0.5 (−4.0 to 0.5)**	< 0.001
ΔHb ^a^ (g/dL)	–0.3 (–1.4 to 0.9)	0.1 (–1.3 to 0.8)	–0.2 (–1.7 to 0.7)	–0.4 (−0.4 to 1.4)	0.37
ΔAlb ^a^ (g/dL)	0.1 (–0.1 to 0.2)	0 (−0.2 to 0.1)	−0.1 (−0.3 to 0.1)**	−0.2 (−0.4 to 0)**	< 0.001
ΔcCa^a^ (mg/dL)	−0.7 (−0.1 to 0.4)	0.1 (−0.4 to 0.6)	0.1 (−0.4 to 0.7)	0.2 (−0.5 to 0.7)	0.09
ΔP^a^ (mg/dL)	−0.2 (−1.2 to 1.4)	−0.5 (−1.3 to 0.7)	0.05 (−1.5 to 0.9)	−0.4 (−1.5 to 1.0)	0.58
ΔALP^a^ (IU/L)	6 (−42 to 39)	5 (−45 to 40)	−22 (−83 to 13)**	11 (−28 to 84)	0.003
ΔBUN^a^ (mg/dL)	0 (−7 to 11)	−3 (−11 to 9)	−9 (−18 to 1)**	−7 (−23 to 7)*	0.002
ΔCr ^a^ (mg/dL)	−0.1 (−0.8 to 1.3)	0.1 (−1.1 to 1.0)	−0.4 (−1.2 to 0.4)*	−0.7 (−1.9 to 0.4)**	0.01
ΔTC ^a^ (mg/dL)	3 (−13 to 18)	2 (−14 to 17)	−2.5 (−20 to 17)	2 (−13 to 21)	0.87
ΔCRP^a^ (mg/dL)	0 (−0.2 to 0.2)	0 (0 to 0.2)	0 (−0.1 to 0.1)	0 (0 to 1.2)	0.11

Kruskal–Wallis test was used.

*CTR* cardiothoracic ratio, *DW* dry weight, *Hb* hemoglobin, *Alb* albumin, *cCa* corrected calcium, *P* phosphate, *ALP* alkaline phosphatase, *BUN* blood urea nitrogen, *Cr* creatinine, *TC* total cholesterol, *CRP* C-reactive protein.

* vs Q1 p < 0.05.

** vs Q1 p < 0.01.

^a^Median (interquartile range).

According to the multivariable logistic regression model, patients classified into %PMI Q4 (the lowest) were associated with an annual decrease in albumin levels and dry weight. Moreover, %PMI Q4 was also associated with female sex and ECOG-PS 2–4 (vs. PS 0–1) (Table 4). Vitamin D did not have a positive effect on %PMI (p = 0.50). Similarly, another multivariable logistic regression model for %PMI Q4 (the lowest) was performed using the patient’s background at the entry period. However, only female sex and ECOG-PS 0–1 vs. 2–4 were associated with %PMI Q4 (Supplementary Table 3).

**Table 4:** Logistic regression model for Q4 of % change of psoas muscle index.

	Univariate			Multivariable		
	OR	95% CI	P value	OR	95% CI	P value
Age (years)	1.03	1.01–1.06	0.004	1.01	0.98–1.04	0.57
Male vs. Female	0.34	0.20–0.60	< 0.001	0.32	0.17–0.60	< 0.001
Dialysis vintage	1.00	0.97–1.03	0.96			
ΔCTR (%)	0.94	0.87–1.01	0.10			
ΔDW (kg)	0.82	0.75–0.90	0.004	0.87	0.77–0.97	0.01
ΔHb (g/dL)	0.86	0.72–1.01	0.06			
ΔAlb (g/dL)	0.14	0.05–0.34	< 0.001	0.22	0.07–0.67	0.007
ΔcCa (mg/dL)	1.18	0.86–1.61	0.30			
ΔP (mg/dL)	0.91	0.77–1.06	0.23			
ΔALP /10 (IU/L)	1.03	1.01–1.05	0.005	1.02	0.99–1.04	0.13
ΔBUN (mg/dL)	0.99	0.97–1.00	0.10			
ΔCr (mg/dL)	0.81	0.70–0.93	0.003	0.93	0.78–0.97	0.38
ΔTC (mg/dL)	1.00	0.99–1.01	0.95			
ΔCRP (mg/dL)	1.14	1.01–1.28	0.02	0.97	0.83–1.14	0.74
ECOG-PS 0–1 vs. 2–4 (entry)	4.11	2.33–7.27	< 0.001	2.46	1.22–4.93	0.01

*OR* odds ratio, *CI* confidence interval, *CTR* cardiothoracic ratio, *DW* dry weight, *Hb* hemoglobin, *Alb* albumin, *cCa* corrected calcium, *P* phosphate, *ALP* alkaline phosphatase, *BUN* blood urea nitrogen, *Cr* creatinine, *TC* total cholesterol, *CRP* C-reactive protein, *ECOG-PS* Eastern Cooperative Oncology Group performance status.

### Survival analysis

**Figure 3:**
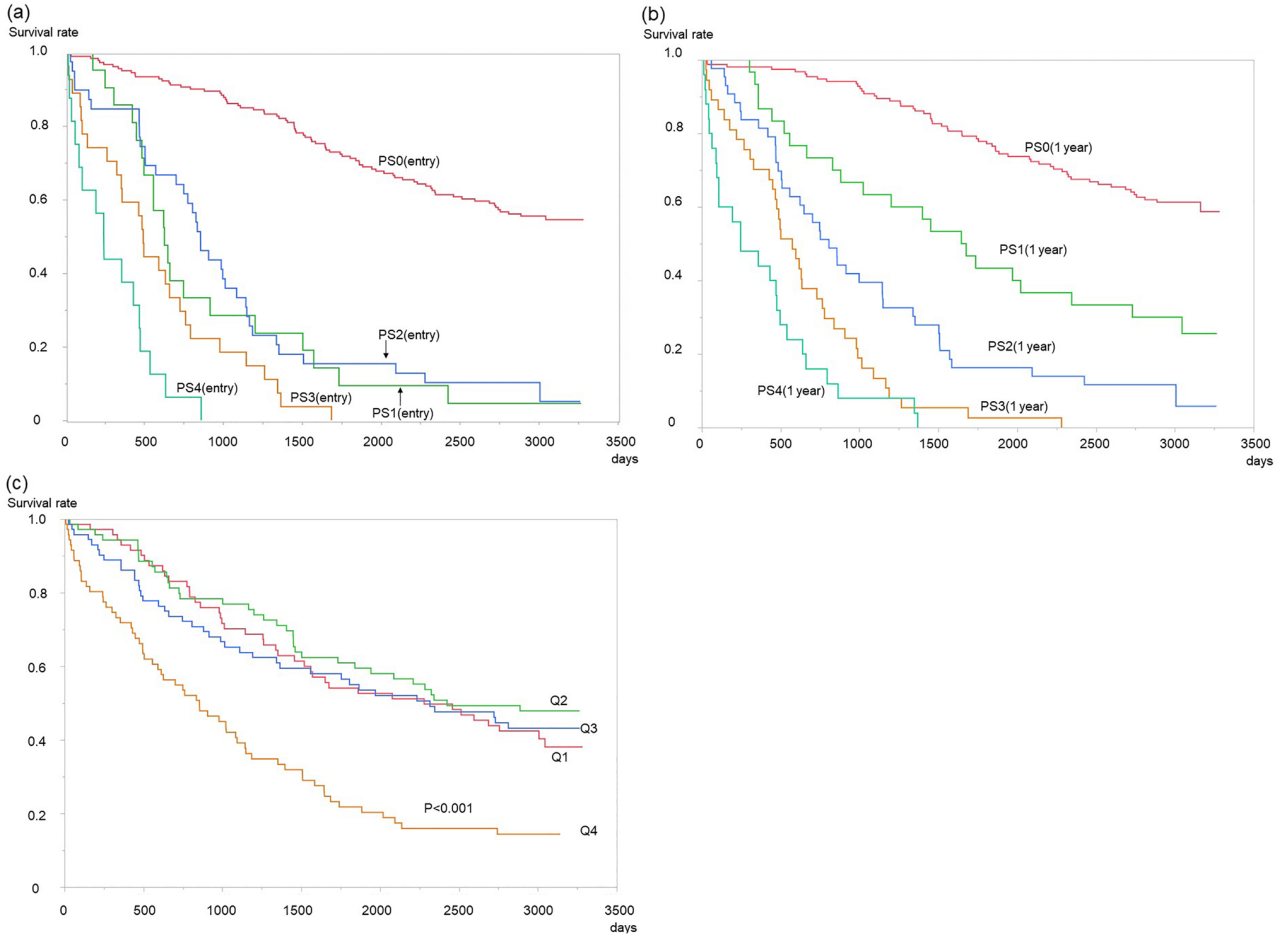
Kaplan–Meier analyses of participants. **(a)** Survival curve by the Eastern Cooperative Oncology Group Performance Status (ECOG-PS) at the entry. PS: Eastern Cooperative Oncology Group Performance Status. Apart from ECOG-PS 1 and PS 2, there were significant differences in each group (p < 0.001). **(b)** Survival curve by the ECOG-PS 1 year after entry. There were significant differences in each group (p < 0.001). **(c)** Survival curve by quartile of % change in the psoas muscle mass index (Q1–Q4). There was no significant difference among Q1–Q3; however, Q4 had a significantly worse outcome compared with Q1–Q3 (p < 0.001). Statistical analyses were performed using the JMP Pro 15.0.0 (3903308).

The median follow-up period was 1561 days (interquartile, 626–2996 days). The survival curves with respect to ECOG-PS (entry) and ECOG-PS (1 year later) indicated that physical function was closely associated with all-cause mortality. Regarding the survival curve with respect to ECOG-PS (entry), there was no significant difference between ECOG-PS 1 and 2; however, the differences between the other groups were statistically significant (Fig. 3A). Contrastingly, there were clear differences among each category of the ECOG-PS (1 year later) (Fig. 3B). %PMI Q4 was associated with a significantly worse outcome, but there was no significant difference among Q1–Q3 (Fig. 3C).

### Cox regression analyses

The multivariable Cox regression models are shown in Table 5. Age, sex, HD vintage, HD time, diabetes history, ischemic heart disease history, stroke history, CTR, dry weight, hemoglobin, serum albumin, corrected calcium, phosphate, serum creatinine, blood urea nitrogen, and C-reactive protein adjusted multivariable Cox proportional analyses indicated that %PMI (model 1) and ECOG-PS (1 year later) (model 2) were significantly associated with the patients’ prognosis; however, if these two parameters were included in the same model (model 3), %PMI was not correlated with all-cause mortality (p = 0.054), although the ECOG-PS was still significantly associated with prognosis. Moreover, age, sex, history of diabetes mellitus, and hemoglobin and albumin levels were consistently correlated with all-cause mortality in all the three models (p < 0.05).

**Table 5:** Multivariable Cox regression analysis for all-cause mortality.

	Model 1			Model 2			Model 3		
	HR	95% CI	P value	HR	95% CI	P value	HR	95% CI	P value
Age (years)	1.03	1.01–1.05	0.004	1.02	1.00–1.04	0.02	1.02	1.00–1.04	0.03
Male vs. Female	2.41	1.61–3.59	< 0.001	1.95	1.30–2.91	0.001	2.14	1.41–3.24	< 0.001
HD vintage /year	1.00	0.98–1.03	0.77	0.99	0.97–1.02	0.52	0.99	0.97–1.02	0.57
HD time /h	0.65	0.45–0.94	0.02	0.81	0.56–1.16	0.24	0.78	0.54–1.13	0.18
IHD history	1.05	0.77–1.45	0.74	1.26	0.91–1.76	0.17	1.19	0.85–1.66	0.31
DM history	1.85	1.32–2.59	0.01	1.59	1.11–2.22	0.01	1.66	1.14–2.30	0.007
Stroke history	1.12	0.79–2.59	0.53	1.06	0.74–1.50	0.76	1.02	0.72–1.46	0.89
CTR/1%	1.03	0.999–1.07	0.051	1.00	0.97–1.04	0.86	0.99	0.97–1.04	0.71
Dry weight /kg	0.99	0.97–1.01	0.21	0.99	0.97–1.01	0.18	0.99	0.97–1.01	0.16
Hb/g/dL	0.82	0.69–0.97	0.02	0.78	0.66–0.92	0.003	0.78	0.66–0.91	0.002
Alb /g/dL	0.33	0.19–0.58	< 0.001	0.46	0.26–0.82	0.009	0.46	0.26–0.82	0.008
cCa /mg/dL	0.92	0.67–1.25	0.58	0.84	0.62–1.15	0.28	0.84	0.62–1.15	0.27
P /mg/dL	0.94	0.83–1.06	0.55	0.96	0.88–1.13	0.96	0.98	0.87–1.12	0.81
BUN /10 mg/dL	1.04	0.92–1.18	0.50	0.99	0.87–1.12	0.87	1.00	0.89–1.15	0.91
Cr / mg/dL	0.89	0.83–0.97	0.006	0.94	0.86–1.02	0.16	0.94	0.86–1.02	0.16
CRP /mg/dL	1.05	0.97–1.13	0.23	1.05	0.97–1.12	0.23	1.04	0.96–1.12	0.35
%PMI change	0.99	0.97–0.995	0.004				0.99	0.98–1.00	0.054
ECOG-PS1 (1 year)				2.10	1.84–5.87	0.007	2.06	1.20–3.51	0.008
ECOG-PS2 (1 year)				2.94	1.20–3.43	< 0.001	2.75	1.63–4.62	< 0.001
ECOG-PS3 (1 year)				5.42	2.46–8.05	< 0.001	5.02	2.79–9.02	< 0.001
ECOG-PS4 (1 year)				7.57	3.16–14.80	< 0.001	7.36	3.78–14.35	< 0.001

Model 1: The predetermined factors and %PMI adjusted; Model 2: The predetermined factors and ECOG-PS adjusted; Model 3: The predetermined factors and %PMI and ECOG-PS adjusted.

*HR* hazard ratio, *CI* confidence interval, *HD* hemodialysis, *IHD* ischemic heart disease, *DM* diabetes mellitus, *CTR* cardiothoracic ratio, *DW* dry weight, *Hb* hemoglobin, *Alb* albumin, *cCa* corrected calcium, *P* phosphate, *BUN* blood urea nitrogen, *Cr* creatinine, *TC* total cholesterol, *CRP* C-reactive protein, *ECOG-PS* Eastern Cooperative Oncology Group performance status.

## Discussion

We investigated the prognosis of patients receiving HD in a single center and observed that both %PMI and physical function were significantly correlated with their prognosis, and that %PMI was associated with their physical function, particularly ECOG-PS 2 and 3. The lowest quartile of %PMI (Q4) (< −20.5%) showed extremely poor prognosis.

Sarcopenia is defined as a decrease in the volume and strength of skeletal muscles^11^. However, there are two types of sarcopenia: aging-associated sarcopenia and secondary sarcopenia^2^. The latter is more intense and occurs on a larger scale compared with natural aging-induced muscle mass loss. Natural aging deteriorates muscle mass by approximately 1–2% annually^12–14^; however, patients with high comorbidity lose their muscle mass rapidly^15^. For example, patients with esophageal cancer had decreased muscle mass (~ 18%) from the initial visit to 3 months after neoadjuvant chemoradiotherapy and esophagectomy^16^.

Chronic kidney disease is one of the most important etiologies of secondary sarcopenia, especially when HD is required. Although some studies have shown that lean body mass loss was not marked in patients undergoing HD^17–19^, a previous report showed that patients receiving HD who could not receive kidney transplantation due to comorbidities had a decrease in lean body mass by 17.1% in 20 weeks^20^. In this study, the participants receiving HD had a mean age of approximately 70 years, and their median annual %PMI was −9.5%. Moreover, we included not only outpatients but also inpatients and nursing home residents on maintenance HD. Some of the patients had other complications, such as cardiovascular diseases. Considering previously reported results, this study indicated that the decreasing rate of muscle mass in patients receiving HD varies depending on the situation. Nonetheless, the muscle mass loss in HD populations will be greater than that in the general population at the same age.

According to the diagnostic criteria proposed by the Asian Working Group for Sarcopenia^21^, the analysis of muscle mass using dual-energy X-ray absorptiometry, handgrip strength, and gait speed is needed to diagnose sarcopenia. Although it is relatively easy to diagnose sarcopenia using these diagnostic criteria, it remains difficult to use them to retrospectively evaluate patient characteristics. Moreover, bioelectrical impedance analysis is not readily available in HD facilities for assessing skeletal muscles^7^. The PMI is known to reflect the total volume of skeletal muscle and predict complications and prognosis after surgery^7,8,22,23^, and calculating the PMI is relatively easy and less time-consuming^24^. Furthermore, previous studies have shown that the PMI has good reliability, including intra-rater reliability and inter-rater reliability^7,10^. Thus, several studies have recently investigated the PMI in patients receiving HD^7–9^ and clarified that the PMI correlated with their long-term survival after cardiac surgery^8^ and the erythropoietin response^9^. Contrastingly, few studies have investigated annual PMI changes in patients receiving HD. In this study, %PMI had a negative impact on prognosis, particularly in the lowest quartile (Q4, < −20.5%) group. A previous report on patients with esophageal cancer showed almost the same tendencies; those with larger decreases in the PMI (< 20%) had poor prognoses^16^.

We adopted the ECOG-PS as an evaluator of physical function in patients receiving HD. Although this scale is widely used in the medical field, only a few reports have used it to evaluate physical function in patients receiving HD^25,26^. Since this study was based on medical records, we could not use more detailed scales to evaluate physical function, such as the Minimum Data Set Activities of Daily Living scale^27^ and the Functional Independence Measure score^28^. Nevertheless, the ECOG-PS was a strong predictor of patient survival and was closely associated with %PMI.

Incidentally, the multivariate Cox regression analysis adjusting 1 year after the physical function did not show that %PMI was associated with all-cause mortality (p = 0.054); this might have been due to the collinearity of %PMI and physical function. The medians of %PMI decreased in accordance with the performance status (Supplementary Fig. 2). Nonetheless, accumulating evidence shows that muscle function decline precedes muscle mass decrease^1,2^, and that muscle function is more important for prognosis. Therefore, the significance of physical function in prognosis may outweigh that of muscle mass loss.

The precise mechanisms of sarcopenia among patients receiving HD have not been fully elucidated, although several factors have been suggested^1,2^. First, uremic toxins such as indoxyl sulfate evoke oxidative stress and insulin resistance, resulting in protein energy wasting^29^. Uremic toxins and inorganic phosphate have been proven to hinder myogenic differentiation of skeletal muscle cells^2^. Second, chronic kidney disease-mineral bone disorders (CKD-MBD) influence muscle atrophy. Vitamin D deficiency, higher phosphate levels, and parathyroid hormone negatively affect muscle mass. For instance, 1α, 25-dihydroxyvitamin D is known to promote myogenic differentiation via vitamin D receptors^2^. Third, malnutrition and amino acid loss during HD accelerate muscle mass loss^30^. Finally, physical inactivity and a sedentary lifestyle in patients receiving HD also contribute to sarcopenia^31^. In this study, the serum creatinine, blood urea nitrogen, and CKD-MBD parameters were not associated with the results of the multivariable logistic regression analysis for %PMI (Q4); however, female sex, serum albumin, and physical function had a greater impact on muscle mass loss. A previous report has shown that Asian patients receiving HD are vulnerable to muscle mass loss^32^, although the differences in sex for muscle mass loss remained controversial; however, another report showed no significant differences in sex in terms of sarcopenia in Japanese patients receiving HD^3^. Therefore, our data cannot be generalized to other populations. Vitamin D did not have a significant effect on %PMI in this study. This means that the importance of nutrition and physical functions outweighs HD conditions and treatment for CKD-MBD in counteracting muscle mass loss. Notably, malnutrition has been known to cause sarcopenia^33^.

As shown in the logistic regression model for %PMI (Q4), %PMI was closely associated with Δ serum albumin, indicating that poor nutritional status accelerates muscle mass loss. Thus, nutritional intervention could improve the sarcopenic status in patients receiving HD. Previous studies have shown that supplementation with essential amino acids, especially leucine, could improve muscle mass^34^, and that medium-chain triglycerides can improve muscle strength and function^35^. Further studies should be conducted to elucidate the types of nutrition and substances that can improve muscle mass and muscle strength of patients receiving HD.

Physical function strongly predicts life prognosis in patients receiving HD^27^, and their physical function changes rapidly when they undergo surgery or hospitalization^36^. Even 1 week of bed rest during hospitalization causes considerable muscle mass loss (3.2% decrease in the cross-sectional area of the quadriceps muscle) and insulin resistance in the whole body^37^, which accelerate the vicious cycle of sarcopenia. In this study, patients who were categorized as ECOG-PS 2 and 3 experienced a rapid decrease in muscle mass; this means that physical function affects muscle mass, and we should consider interventions such as resistance training and multimodal exercise^38^, and avoid iatrogenic disability through long-term hospitalization^39^, if possible. Notably, medical staff may also contribute to the sedentary lifestyle of patients receiving HD; for example, they may be reluctant to recommend exercise^40^. Although the %PMI in patients with ECOG-PS 0–3 decreased accordingly, patients with ECOG-PS 4 did not show a rapid decrease in PMI. We speculated that these patients were treated as in-hospital patients and were served appropriate meals or nutrition, which might have prevented muscle mass loss compared with patients with ECOG-PS 2–3.

Compared with the ECOG-PS, although the PMI or %PMI is not a strong tool to predict patients’ prognosis, the PMI is a simple and useful tool for evaluating muscle mass in patients receiving HD in clinical practice and can, therefore, be used in various future studies. Furthermore, the %PMI can reflect the change in muscle mass during a certain period. For example, it is easy to detect malnutrition in patients who need intensive nutritional intervention. Additionally, the PMI would be effective in motivating patients to improve their physical function. Visualization of the patient's muscle mass can be a good opportunity for the patient to be involved in exercise therapy and nutritional supplementation. Although patients in our facility do not receive exercise programs during HD sessions, a previous report has shown that exercise training during HD sessions has a positive impact on muscle mass ^41^.

This study has several limitations. First, this study was conducted in a single center; therefore, the reliability of the data in this study is not guaranteed. Patients in our facility might have had various comorbidities as our facility accepts hospitalized patients, and this might have affected our results. Moreover, in addition to outpatients, inpatients and patients who lived in nursing homes were included in this study, as long as their CT images were available; the %PMI in these individuals might have been higher than that in patients receiving HD in other facilities. Additionally, the patient transfer service for every HD session in our facility might have had a negative effect on muscle mass. Moreover, almost all the patients included in this study were Japanese, and their characteristics might differ from those of patients in other countries. Validation studies in other facilities will be needed in the future. Second, we could not evaluate the status of sarcopenia properly as the PMI and ECOG-PS were the only alternative methods. Owing to the retrospective nature of the study, other aspects of sarcopenia, such as handgrip strength and 6-min walk, could not be evaluated. In other words, it was impossible for us to evaluate muscle strength retrospectively. Therefore, we could not compare the muscle mass and muscle strength in this study population. Third, this study was conducted based on the annual screening CT for renal cell carcinoma and other malignancies. Although regular screening by abdominal ultrasonography and CT examination may be useful for young people and patients undergoing long-term dialysis^42,43^, there is no concrete evidence supporting the requirement of annual CT screening in patients receiving HD. Furthermore, %PMI, which we focused on in this study, can only be obtained through more than two sessions of CT, which implies that %PMI is only available in a retrospective manner. Therefore, the significance of annual CT screenings and obtaining the %PMI may be limited.

In conclusion, the annual muscle mass loss in patients receiving HD was significantly associated with physical function, and annual change in serum albumin levels and dry weight. In addition to higher ECOG-PS, patients with extensively decreased muscle mass (%PMI < –20%) showed the poorest prognosis. Therefore, consideration should be given to patients with deteriorated physical function, including patients with ECOG-PS 2 and 3, and attempts should be made to maintain their muscle mass. Further, patients receiving HD in whom muscle mass deterioration is quicker should be identified using %PMI to improve their prognosis.

## Methods

### Patients

We included patients receiving HD who were treated at the Nagasaki Renal Center between July 2011 and June 2013. The inclusion criterion was as follows: patients aged ≥ 20 years with a dialysis duration of > 3 months. The exclusion criteria were as follows: patients who left the Nagasaki Renal Center or died during the inclusion period (July 2011–July 2013) and did not undergo annual screening examinations, including abdominal CT (BrightSpeed; GE Healthcare, Chicago, USA), during their birthday months. The patient flowchart is shown in Supplementary Fig. 1. The observation period commenced immediately after the patients’ birthdays, from July 2012 to June 2013, and finally, to June 2021.

### Data collection

Patient characteristics, such as age, sex, duration of dialysis, blood examinations, complications, and drug prescription history were obtained from medical records between July 2011 and June 2013. Two data points corresponding to each patient’s birthday were used. Blood pressure was measured immediately before the HD session. A ratio of 1:200 was used to convert darbepoetin alfa and epoetin beta pegol to epoetin alfa, as described previously^44^. The PMI was calculated as previously described^8–10^. Briefly, at the edge of the lower third lumbar vertebra, the transverse area of the psoas muscle was traced manually using Synapse (Fuji Film, Tokyo, Japan). Two observers independently calculated the area of the psoas muscle (M.K., K.Y.) after being trained by a radiologist. Owing to the availability of annual routine abdominal CT, the PMI was analyzed at two time points: from July 2011 to June 2012 (entry time), and from July 2012 to June 2013 (1 year later). %PMI was calculated as follows: [PMI (1 year later) − PMI (entry time)]/PMI (entry time) × 100. To identify the factors affecting %PMI, blood examination and drug information was collected during their birth months from July 2011 to July 2012. The physical function in patients receiving HD was evaluated using the ECOG-PS scale, as described previously^25,26^. Since ECOG-PS ≥ 2 was significantly associated with sarcopenia in Japanese patients^45^, we divided the patients according to ECOG-PS 0–1 and 2–4. Two aspects of the PMI were analyzed from July 2011 to June 2012 (entry time) and July 2012 to June 2013 (1 year later) from the medical records, namely, the degree of nursing care necessity in their daily lives, and ambulatory status to receive in-center HD. The evaluation was performed by M.K. and K.Y. independently. If there was a discrepancy, the two observers reevaluated the patient’s status.

### Statistical analyses

Continuous values are presented as mean ± standard deviation. For non-normally distributed data, median with interquartile values are shown. Categorical values are shown as numbers (%) and continuous variables as mean ± standard deviation. The participants were divided into four groups according to the quartile of %PMI. The paired or non-paired t-test and Wilcoxon sum rank test were used to compare the continuous variables between the two groups. The Chi-squared test or McNemar test was used to compare the categorical variables. The one-way analysis of variance or the Kruskal–Wallis test was used to compare more than three groups. A logistic regression analysis was conducted to elucidate the association between %PMI Q4 (the lowest) and patient backgrounds. Among age, sex, dialysis vintage, ECOG-PS (entry) 0–1 vs. 2–4, and other annual changes in blood examination, parameters with significant levels of p-values in the univariate logistic regression analysis were included in a multivariate logistic regression model. The Kaplan–Meier curves were used to show the survival rate for some groups, and the log-rank test was used to compare the groups. The multivariable Cox regression models were used to evaluate the risk of mortality, and age, sex, HD vintage, HD time, diabetes history, ischemic heart disease history, stroke history, CTR, dry weight, hemoglobin, serum albumin, corrected calcium, phosphate serum creatinine, blood urea nitrogen, and C-reactive protein were included in models 1–3. Model 1 included %PMI, model 2 included the ECOG-PS, and model 3 included %PMI and the ECOG-PS, in addition to the factors stated above. Statistical significance was set at p < 0.05. Statistical analyses were performed using the JMP Pro 15.0.0 (3903308) (SAS Institute Inc., Cary, NC, USA. https://www.jmp.com/en_my/software/new-release/new-in-jmp-and-jmp-pro.html).

### Ethical approval

This study was approved by the Nagasaki Renal Center Clinical Research Ethics Committee (Nagasaki Japan) (21006) and adhered to the 1964 Declaration of Helsinki and subsequent amendments. The included patients were informed, although obtaining consent was not required as this was a retrospective analysis, and the patient data were anonymized. The ethics committee approved the waiver of informed consent.

## Supplementary Information

Supplementary Figure S1.

Supplementary Figure S2.

Supplementary Table S1.

Supplementary Table S2.

Supplementary Table S3.

## Data Availability

The datasets analyzed during the current study are available from the corresponding author upon reasonable request.
