# Supplementary material for: The impact of muscle mass loss and deteriorating physical function on prognosis in patients receiving hemodialysis
Source: Sci Rep. 2021 Nov 16;11:22290. doi: 10.1038/s41598-021-01581-z (PMC8595648; doi:10.1038/s41598-021-01581-z)
Supplement: Supplementary file 1 — Supplementary Figure S1. [file 41598_2021_1581_MOESM1_ESM.pptx]

## Slide 1
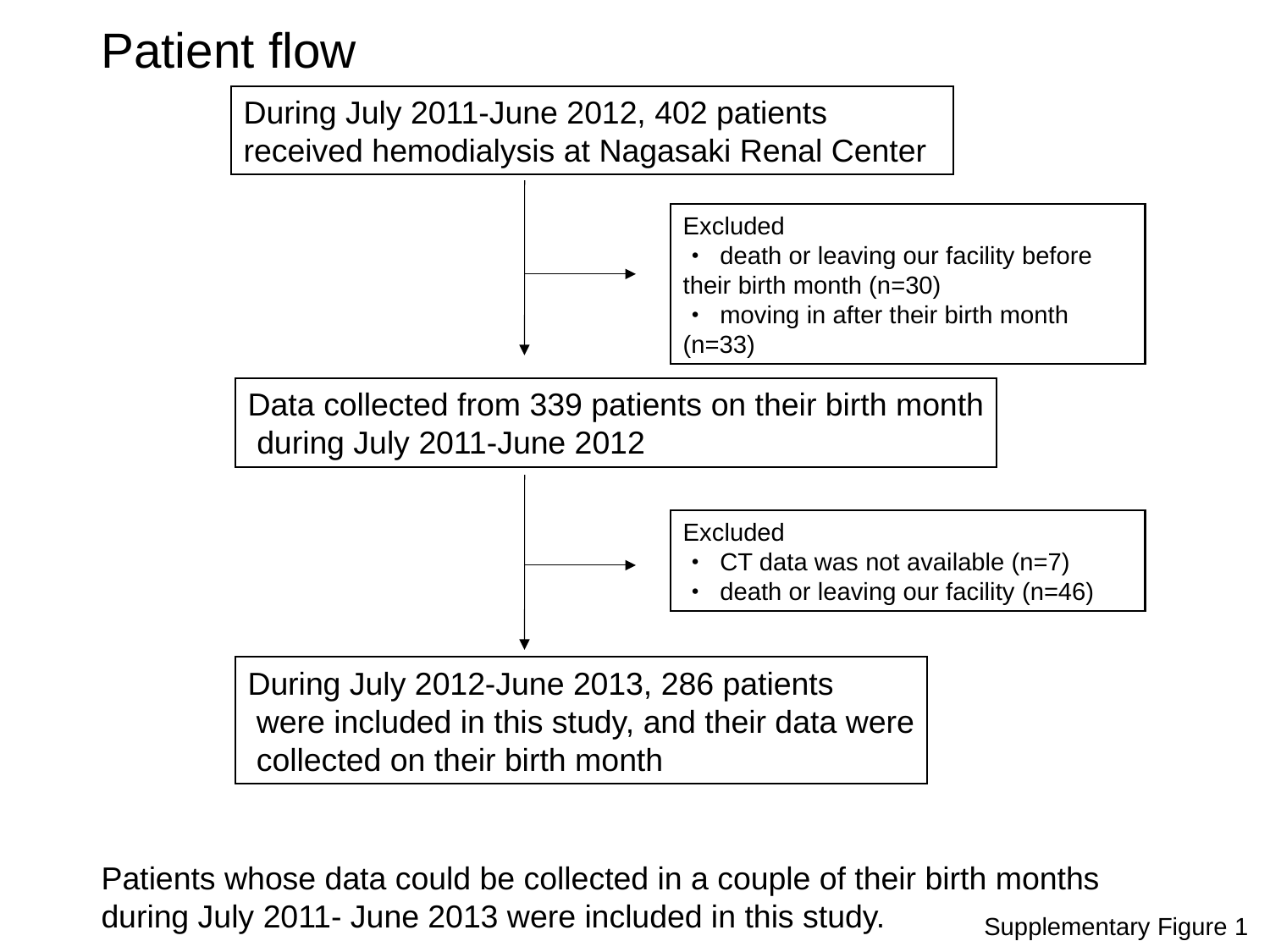

Patient flow
During July 2011-June 2012, 402 patients received hemodialysis at Nagasaki Renal Center
Excluded
・ death or leaving our facility before their birth month (n=30)
・ moving in after their birth month (n=33)
Data collected from 339 patients on their birth month
 during July 2011-June 2012
Excluded
・ CT data was not available (n=7)
・ death or leaving our facility (n=46)
During July 2012-June 2013, 286 patients
 were included in this study, and their data were
 collected on their birth month
Patients whose data could be collected in a couple of their birth months during July 2011- June 2013 were included in this study.
Supplementary Figure 1
