# Supplementary material for: The impact of muscle mass loss and deteriorating physical function on prognosis in patients receiving hemodialysis
Source: Sci Rep. 2021 Nov 16;11:22290. doi: 10.1038/s41598-021-01581-z (PMC8595648; doi:10.1038/s41598-021-01581-z)
Supplement: Supplementary file 2 — Supplementary Figure S2. [file 41598_2021_1581_MOESM2_ESM.pptx]

## Slide 1
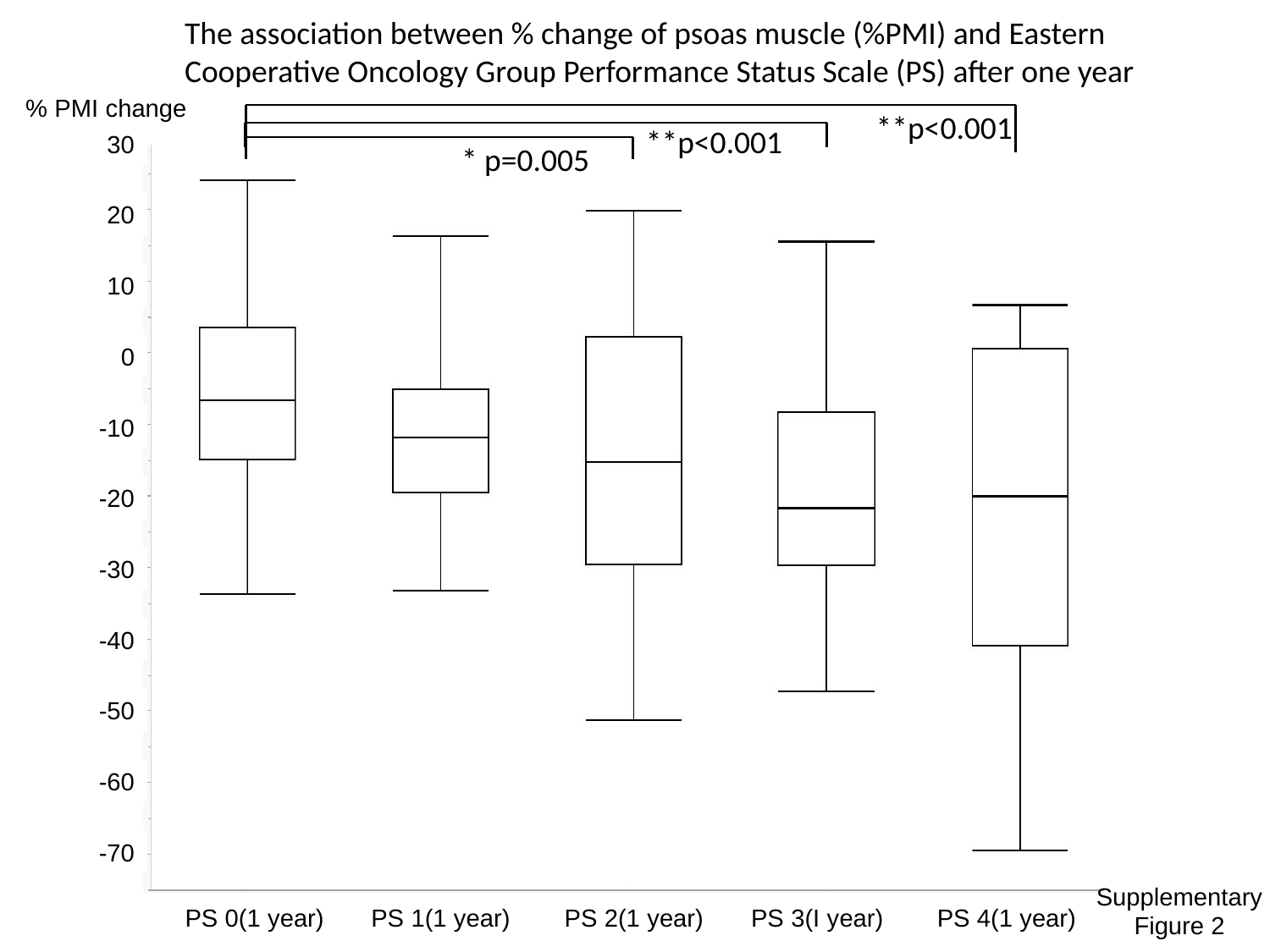

The association between % change of psoas muscle (%PMI) and Eastern Cooperative Oncology Group Performance Status Scale (PS) after one year
% PMI change
**p<0.001
**p<0.001
30
* p=0.005
20
10
0
-10
-20
-30
-40
-50
-60
-70
Supplementary
Figure 2
PS 0(1 year)
PS 1(1 year)
PS 2(1 year)
PS 3(I year)
PS 4(1 year)
