# Supplementary material for: The impact of muscle mass loss and deteriorating physical function on prognosis in patients receiving hemodialysis
Source: Sci Rep. 2021 Nov 16;11:22290. doi: 10.1038/s41598-021-01581-z (PMC8595648; doi:10.1038/s41598-021-01581-z)
Supplement: Supplementary file 3 — Supplementary Table S1. [file 41598_2021_1581_MOESM3_ESM.docx]

**Supplementary Table 1 Patient background (other factors)**

|  | At the time of entry  (N=286) | One year after  (N=286) | P value |
| --- | --- | --- | --- |
| Anti-platelet drugs (%) | 38 | 38 | 1.0 |
| Warfarin (%) | 7 | 10 | 0.008 |
| ESA^a^ (IU/week) | 4000 (2000–8000) | 5000 (2500–10000) | <0.001 |
| Iron (%) | 20 | 13 | 0.02 |
| Calcium carbonate (%) | 50 | 48 | 0.40 |
| Lanthanum carbonate (%) | 34 | 30 | 0.10 |
| Sevelamer (%) | 4 | 10 | <0.001 |
| Cinacalcet (%) | 18 | 19 | 0.87 |
| Vitamin D (%) | 70 | 73 | 0.29 |

ESA, erythropoiesis-stimulating agents
