# Supplementary material for: The impact of muscle mass loss and deteriorating physical function on prognosis in patients receiving hemodialysis
Source: Sci Rep. 2021 Nov 16;11:22290. doi: 10.1038/s41598-021-01581-z (PMC8595648; doi:10.1038/s41598-021-01581-z)
Supplement: Supplementary file 4 — Supplementary Table S2. [file 41598_2021_1581_MOESM4_ESM.docx]

**Supplementary Table 2. The association between the quartile of psoas mass index and patient background**

|  | Q1  (n=71) | Q2  (n=72) | Q3  (n=72) | Q4  (n=71) | P  value |
| --- | --- | --- | --- | --- | --- |
| Age (years) | 66.7±12.4 | 62.3±15.0 | 64.7±12.5 | 69.6±11.0 | 0.007 |
| Male (%) | 62.0 | 63.9 | 66.7 | 38.0 | 0.002 |
| Dialysis vintage^a^ (months) | 67 (23–126) | 63 (25–154) | 63 (29–122) | 48 (21–120) | 0.91 |
| CTR (%) | 51±6 | 51±5 | 51±5 | 53±6 | 0.06 |
| Dry weight (kg) | 53.9±8.7 | 53.7±12.7 | 54.1±10.7 | 50.6±10.4 | 0.16 |
| BMI (kg/m^2^) | 21.3±2.8 | 21.0±3.8 | 21.1±3.3 | 21.0±3.3 | 0.97 |
| Diabetes (%) | 31 | 35 | 33 | 37 | 0.91 |
| IHD (%) | 28 | 35 | 39 | 35 | 0.59 |
| Stroke (%) | 30 | 22 | 15 | 37 | 0.02 |
| sBP (mmHg) | 150±23 | 152±24 | 153±22 | 145±27 | 0.21 |
| Hb (g/dL) | 10.7±1.5 | 10.9±1.3 | 11.0±1.3 | 10.8±1.4 | 0.68 |
| Alb (g/dL) | 3.5±0.4 | 3.6±0.4 | 3.7±0.4 | 3.5±0.4 | 0.02 |
| cCa (mg/dL) | 9.3±0.8 | 9.2±0.6 | 9.1±0.7 | 9.2±0.7 | 0.14 |
| P (mg/dL) | 5.5±1.5 | 5.5±1.4 | 6.1±1.6 | 5.5±1.7 | 0.03 |
| ALP^a^ (IU/L) | 247 (191–327) | 226 (176–335) | 252 (193–365) | 269 (205–325) | 0.16 |
| BUN (mg/dL) | 64.5±16.5 | 68.6±16.0 | 74.9±14.1 | 68.0±18.2 | 0.002 |
| Cr (mg/dL) | 10.5±3.6 | 10.7±3.2 | 11.5±3.2 | 9.6±3.1 | 0.008 |
| TC (mg/dL) | 161±42 | 161±35 | 160±34 | 162±36 | 0.99 |
| CRP^a^ (mg/dL) | 0.22  (0.07–0.46) | 0.12  (0.05–0.23) | 0.16  (0.07–0.48) | 0.17  (0.08–0.51) | 0.09 |
| KT/V | 1.3±0.5 | 1.4±0.4 | 1.3±0.4 | 1.4±0.4 | 0.60 |

One-way ANOVA (Analysis of Variance) or Kruskal–Wallis test or The Chi-square test was used.

CTR, cardiothoracic ratio; BMI, body mass index; IHD, ischemic heart disease; sBP, systolic blood pressure; Hb, hemoglobin; Alb, albumin; cCa, corrected calcium; P, phosphate; ALP, alkaline phosphatase; BUN, blood urea nitrogen; Cr, creatinine; TC, total cholesterol; CRP, C-reactive protein

^a^ median (interquartile range)
