# Supplementary material for: The impact of muscle mass loss and deteriorating physical function on prognosis in patients receiving hemodialysis
Source: Sci Rep. 2021 Nov 16;11:22290. doi: 10.1038/s41598-021-01581-z (PMC8595648; doi:10.1038/s41598-021-01581-z)
Supplement: Supplementary file 5 — Supplementary Table S3. [file 41598_2021_1581_MOESM5_ESM.docx]

**Supplementary Table3. Logistic regression model for Q4 of % change of psoas muscle index**

|  | Univariate | | | Multivariable | | |
| --- | --- | --- | --- | --- | --- | --- |
|  | OR | 95% CI | P value | OR | 95% CI | P value |
| Age (years) | 1.03 | 1.01–1.06 | 0.004 | 1.01 | 0.98–1.04 | 0.34 |
| Male vs. Female | 0.34 | 0.20–0.60 | <0.001 | 0.32 | 0.15–0.65 | 0.001 |
| Dialysis vintage/year | 1.00 | 0.97–1.03 | 0.96 |  |  |  |
| CTR (%) | 1.07 | 1.02–1.12 | 0.008 | 1.01 | 0.95–1.07 | 0.83 |
| Dry weight (kg) | 0.97 | 0.94–1.00 | 0.02 | 1.02 | 0.98–1.05 | 0.29 |
| BMI (kg/m^2^) | 0.99 | 0.91–1.07 | 0.80 |  |  |  |
| Diabetes (%) | 1.17 | 0.67–2.05 | 0.58 |  |  |  |
| IHD (%) | 1.06 | 0.60–1.86 | 0.85 |  |  |  |
| Stroke (%) | 2.01 | 1.13–3.59 | 0.02 | 2.01 | 1.13–3.59 | 0.07 |
| sBP /10 mmHg | 0.89 | 0.79–1.00 | 0.04 | 0.90 | 0.80–1.01 | 0.07 |
| Hb /g/dL | 0.96 | 0.79–1.17 | 0.72 |  |  |  |
| Alb /g/dL | 0.57 | 0.29–1.11 | 0.10 |  |  |  |
| cCa /mg/dL | 1.07 | 0.73–1.56 | 0.74 |  |  |  |
| P /mg/dL | 0.91 | 0.76–1.09 | 0.29 |  |  |  |
| ALP/ 10IU/L | 1.01 | 0.99–1.03 | 0.30 |  |  |  |
| BUN /mg/dL | 0.95 | 0.81–1.12 | 0.55 |  |  |  |
| Cr /mg/dL | 0.88 | 0.81–0.96 | 0.003 | 1.00 | 0.90–1.12 | 0.95 |
| TC/ 10mg/dL | 1.01 | 0.94–1.09 | 0.82 |  |  |  |
| CRP^/^mg/dL | 1.05 | 0.91–1.22 | 0.51 |  |  |  |
| KT/V | 1.46 | 0.77–2.75 | 0.25 |  |  |  |
| ECOG-PS 0–1 vs. 2–4  (entry) | 4.11 | 2.33–7.27 | <0.001 | 3.24 | 1.63–6.44 | <0.001 |

OR, odds ratio; CI, confidence interval; CTR, cardiothoracic ratio; BMI, body mass index; IHD, ischemic heart disease; sBP, systolic blood pressure; Hb, hemoglobin; Alb, albumin; cCa, corrected calcium; P, phosphate; ALP, alkaline phosphatase; BUN, blood urea nitrogen; Cr, creatinine; TC, total cholesterol; ECOG-PS, Eastern Cooperative Oncology Group Performance Status
